# Anti-Contactin-1-Associated Membranous Nephropathy Presenting With Subsequent Autoimmune Nodopathy

**DOI:** 10.1016/j.xkme.2026.101478

**Published:** 2026-07-18

**Authors:** Ruben Visch, Shoko Vos, Pierre Ronco, Hanna Debiec, Karlien Mul, Tom Nijenhuis, Anne-Els van de Logt

**Affiliations:** 1 Department of Nephrology, Donders Institute for Brain, Cognition and Behaviour, Research Institute for Medical Innovation, Radboud University Medical Center, Nijmegen, The Netherlands; 2 Department of Pathology, Donders Institute for Brain, Cognition and Behaviour, Research Institute for Medical Innovation, Radboud University Medical Center, Nijmegen, The Netherlands; 3 Department of Neurology, Donders Institute for Brain, Cognition and Behaviour, Research Institute for Medical Innovation, Radboud University Medical Center, Nijmegen, The Netherlands; 4 Sorbonne Université, Paris, France; 5 Institut National de la Santé et de la Recherche Médicale, Unité Mixte de Recherche S 1155, Paris, France; 6 Division of Nephrology, Centre Hospitalier du Mans, Le Mans, France

**Keywords:** Membranous nephropathy, contactin-1, autoimmune nodopathy, immunoglobulin G type 4

## Abstract

A 70-year-old man presented with biopsy-proven phospholipase A2 receptor--negative membranous nephropathy (MN). Shortly thereafter, he presented with progressive sensorimotor polyneuropathy, initially consistent with an inflammatory demyelinating polyneuropathy. Laboratory testing revealed strongly positive anti-contactin-1 (CNTN1) autoantibodies, a rare antigen target recently implicated in a subset of MN patients with concurrent inflammatory demyelinating polyneuropathy, reclassifying it as an autoimmune nodopathy. Intensive immunosuppressive treatment including methylprednisolone, cyclophosphamide, plasmapheresis, intravenous immunoglobulins, and rituximab led to significant neurological improvement and seronegativity for anti-CNTN1 antibodies. Nephrotic syndrome improved gradually with partial remission observed 2 years after treatment initiation. This case highlights the autoimmune overlap of MN and autoimmune nodopathy mediated by anti-CNTN1 antibodies, targeting shared epitopes in podocytes and the paranodal region of myelinated peripheral nerves. It underscores the clinical importance of screening for novel antibodies in phospholipase A2 receptor--negative MN patients, particularly anti-CNTN1 when neurological symptoms develop. This report supports aggressive immunosuppression tailored to antibody-mediated disease and suggests anti-CNTN1 titers as a biomarker for monitoring therapeutic response.

## Introduction

Membranous nephropathy (MN) is the most common cause of nephrotic syndrome in adults in the White population.^1^ The disease is characterized by subepithelial deposition of immune complexes on the glomerular basement membrane. Historically, MN has been classified as either primary or secondary to well-defined clinical entities. The vast majority of these secondary causes include infections, malignancies, exposure to specific drugs and toxins, or autoimmune diseases.

The discovery of circulating autoantibodies targeting the phospholipase A2 receptor (PLA2R) in about 70% of MN patients was a major breakthrough. This has significantly advanced the understanding of the disease, confirming that the disease is autoimmune in nature.^2^ Since then, many other autoantigens have been described, questioning the primary versus secondary classification.

The simultaneous occurrence of MN and inflammatory demyelinating poly(radiculo)neuropathy has been reported in several case reports before the identification of a shared target antigen.^3^, ^4^, ^5^ Inflammatory demyelinating polyneuropathy is an acquired immune-mediated disorder affecting the peripheral nerves, classically characterized by symmetrical proximal and distal muscle weakness and sensory involvement.^6^ Several autoantibodies against paranodal proteins have been identified in patients presenting with a chronic inflammatory demyelinating polyneuropathy--like phenotype, including anti-contactin-1 (CNTN1).^7^ CNTN1, a neural adhesion protein of the immunoglobulin (Ig) superfamily, is expressed in glomerular podocytes and has recently been described as a novel antigenic target in a subset of patients with MN.^8^^,^^9^ Autoantibody-mediated neuropathies associated with antibodies targeting nodal and paranodal cell adhesion molecules are now recognized as “autoimmune nodopathies,” distinct from classical chronic inflammatory demyelinating polyneuropathy owing to their specific clinical and electrophysiological features.^6^ Anti-CTN1--associated MN is rare and typically presents with features of nephrotic syndrome, similar to other forms of MN. However, it appears to be associated with specific clinical features, including a higher prevalence in older adults and potential neurological involvement, given the expression of CNTN1 in the peripheral nervous system.^8^

## Case Report

**Figure 1:**
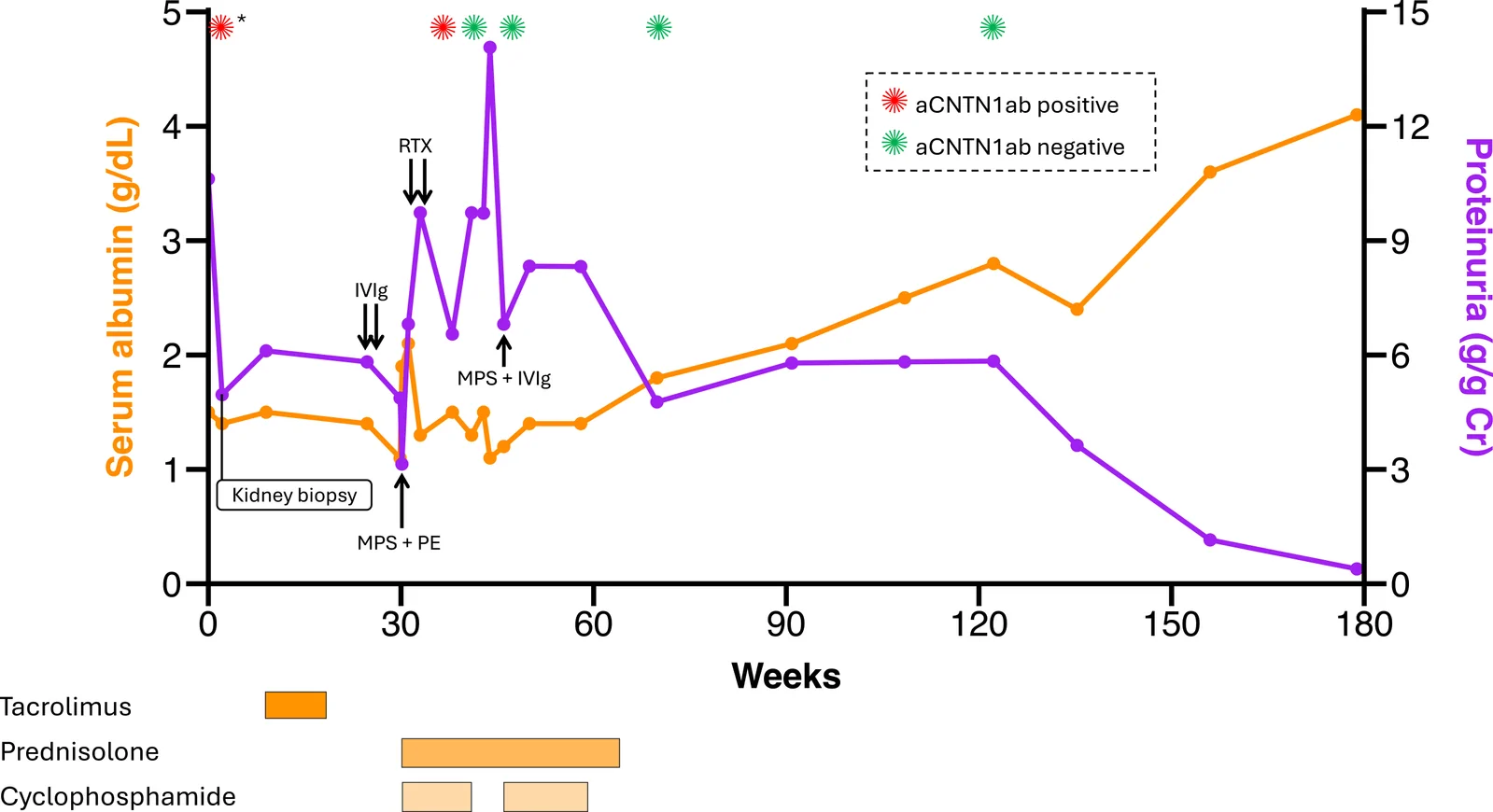
Clinical course and treatment. Abbreviations: aCNTN1ab, anti-contactin-1 antibody; Cr, creatinine; IVIg, intravenous immunoglobulin; MPS, methylprednisolone; PE, plasma exchange; RTX, rituximab. ∗The result of this anti-CNTN antibody test became available around week 30.

A 70-year-old man, with no significant medical history, first presented in June 2022 with progressively worsening lower-limb edema. Laboratory tests revealed nephrotic syndrome (Fig 1). Testing for serum anti-PLA2R antibodies was negative.

**Figure 2:**
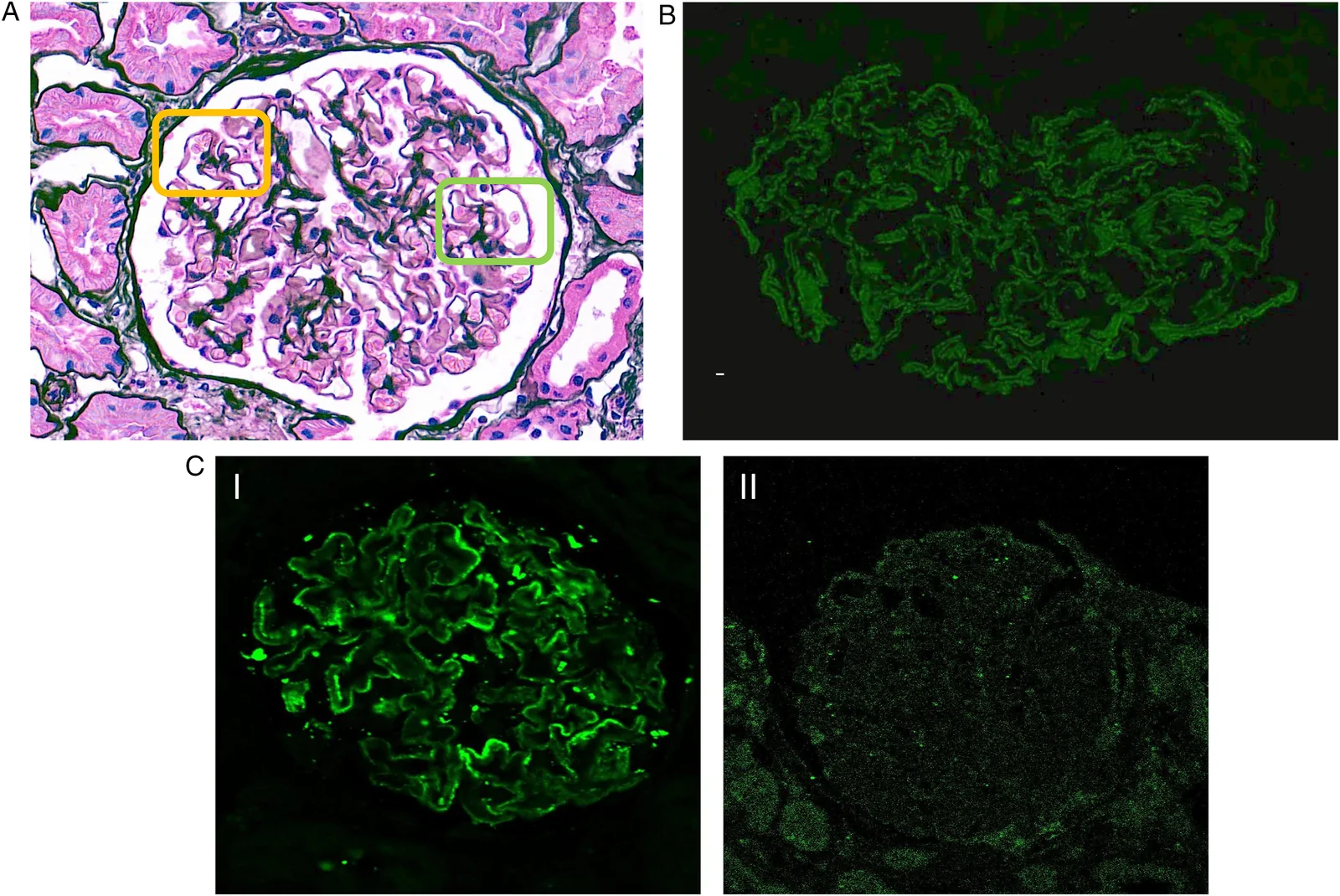
Kidney biopsy findings. (A) Light microscopy (hematoxylin and eosin) of a glomerulus with subtle glomerular basement membrane irregularities with a fenestrated aspect (green outline) and subepithelial eosinophilic deposits (orange outline). (B) Immunofluorescence microscopy showing a glomerulus with a positive immunoglobulin G4 staining (2+) with granular peripheral deposits. (C) Renal specimens from the present case revealing a positive, granular staining (yellow arrows) of CNTN1 antigen along the glomerular basement membrane (I); a sample from a membranous nephropathy patient without neurological disease was used as a negative control (II).

A kidney biopsy was performed, revealing irregularities of the glomerular basement membrane, with a fenestrated aspect and focal spikes in light microscopy (Fig 2A). Immunofluorescence showed a granular pattern for IgG (3+) and C3c (2+) along the glomerular basement membrane, in which IgG4 was the dominant subclass found in the deposits with only minor IgG1 expression (Fig 2B). Electron microscopy revealed diffuse (>80%) foot process effacement and extensive subepithelial depositions, confirming stage 2 MN. PLA2R immunohistochemical staining was negative.

An extensive work-up for secondary causes of MN was negative.

The patient was referred to the Radboudumc Center of Expertise for Rare Kidney Disorders for expert consultation. Despite optimal conservative treatment, his nephrotic syndrome remained severe. Prognostic urinary markers indicated a moderate-to-high risk of developing kidney damage as follows: urinary excretion of alpha-1-microglobulin was elevated at 78 μg/min (cut-off < 50 μg/min) and IgG excretion was elevated at 308 mg (cut-off < 250 mg/24 h), whereas beta-2-microglobulin remained below the cut-off at 618 ng/min (cut-off < 1,000 ng/min).^10^ Given the severity of his clinical symptoms, tacrolimus monotherapy 0.1 mg/kg/d was recommended and subsequently started at the referring center.

The patient had a history of stable numbness in his soles and forefoot for over 10 years, for which he had never sought medical attention. After the start of tacrolimus, the patient developed progressively worsening symptoms of numbness in the feet and legs, which later extended to the hands and fingertips. The patient felt unheard within the conventional medical system. After 6 weeks, he discontinued all medications on his own initiative, including tacrolimus.

In December 2022, the patient was admitted to the emergency department of the referring hospital because of progressive subacute sensorimotor polyneuropathy since 3 months (Item S1). Nerve ultrasound demonstrated multifocal nerve enlargement involving upper limb nerves and the brachial plexus, consistent with an inflammatory demyelinating neuropathy. Nerve conduction studies fulfilled the European Academy of Neurology/Peripheral Nerve Society 2021 criteria for a demyelinating polyneuropathy, based on prolonged distal motor latencies in the ulnar nerve, reduced motor conduction velocities in the median and ulnar nerves bilaterally, and F-wave latencies exceeding 20% above the upper limit of normal. In addition, there was evidence of severe, diffuse sensorimotor axonal loss (Item S2). Treatment with intravenous Ig at 0.4 mg/kg/d was administered twice within 1 month as follows: first for 5 consecutive days, and then again for 2 consecutive days; however, the patient’s neurological condition deteriorated, leading to wheelchair dependency.

The reported association between MN and autoimmune nodopathy prompted us to urgently initiate testing for anti-CNTN1 antibodies. This revealed a strong positive test result for anti-CNTN1 autoantibody presence in his blood in a cell-based assay (Item S3).^11^

In January 2023, the patient was admitted to our hospital to start immunosuppressive treatment aimed at eliminating the suspected anti-CNTN1 autoantibodies. Upon admission, his nephrotic syndrome had worsened, which was as follows: urinary protein-creatinine ratio 4.9 g/g and serum albumin 1.1 g/dL (Fig 1). The Inflammatory Rasch-Built Overall Disability Scale was assessed at 22 (centile metric). He received a regimen consisting of 3 doses of 1,000 mg methylprednisolone on consecutive days, followed by prednisolone 0.5 mg/dg/d every other day, cyclophosphamide (CP) 1.5 mg/kg/d, and 5 sessions of plasmapheresis in 14 days. Two infusions of rituximab 1 g were started after the last session of plasmapheresis. Intravenous furosemide was initiated to manage fluid overload.

One month into treatment, the patient’s neurological symptoms improved significantly. CP was discontinued after 11 weeks owing to leukopenia. In May 2023, owing to worsening of nephrotic syndrome, 3 doses of 1,000 mg methylprednisolone, intravenous Ig 0.4 mg/kg/d for 5 days, and a low-dose CP (50 mg/d) were restarted. However, the decision to restart treatment was made before the results of the April 2023 anti-CNTN1 antibody test were available, which later came back negative for the first time. By June 2023, CP was stopped again as the April 2023 anti-CNTN1 antibody test returned negative for the first time. Neurologically, he showed remarkable recovery and was able to walk independently. However, he developed severe shingles on his chest owing to persistent hypogammaglobulinemia, which was treated with valaciclovir. In addition, by July 2023, he developed erysipelas and received a 4-week course of antibiotics.

At follow-up in April 2024, neurological examination revealed predominantly mild distal muscle weakness and mild gait disturbance (Item S1). The Inflammatory Rasch-Built Overall Disability Scale was assessed at 58 (centile metric). Anti-CNTN1 antibodies remained negative. In June 2025, partial remission of the nephrotic syndrome was observed for the first time (Fig 1).

In retrospect, immunohistochemistry for CNTN1 (serum dilution, 1:100) of the kidney biopsy revealed positive, granular staining along the glomerular basement membrane, confirming MN in anti-CNTN1 antibody--associated autoimmune nodopathy (Fig 2C and Item S3).

## Discussion

This case illustrates a rare yet well-established overlap between MN and autoimmune nodopathy mediated by anti-CNTN1 antibodies, supporting shared autoimmune pathogenesis. Despite diagnostic delay, reinitiation of immunosuppression after CNTN1 antibody detection led to gradual neurological and nephrotic improvement. To our knowledge, a total of 42 patients with CNTN1-associated MN and autoimmune nodopathy have been reported in the literature (Table 1), of which only 2 were pediatric cases. Overall, the mean age of onset was 56.9 years. Of these, 30 were men, 5 were women, and 6 had an unknown sex.

**Table 1:** Characteristics of Patients With MN in Anti-CNTN1 Antibody-Associated CIDP in the Chronological Order of Publication With Available Outcome on Neurology and Nephropathy as Reported in Publication

Case No.	Age (y)	Sex	Uprot	Salb (g/dL)	Scr (mg/dL)	Dominant Anti-CNTN1 Subclass	Stage	Treatment and Response	Reference
1	48	M	NA	NA	NA	IgG4	NA	IVIg: initial improvement; PE: good; CS: transient response	Doppler et al^12^
2	78	M	4.1 g/d	0.7	NA	IgG4	2	CS: renal improvement, no sensorimotor improvement; IVIg: sensorimotor improvement	Hashimoto et al^13^
3	75	M	5.4 g/d	2.2	0.54	IgG4	1/2	CS: improved kidney function, neuropathy worsened; PE: ineffective; RTX: death 1 week after start	Taieb et al^14^
4	58	M	NA	NA	NA	IgG3, IgG4	NA	IVIg: no response; CS: partial response; PE + CP: positive response	Cortese et al^15^
5	43	M	22 g/d	1.5	NA	IgG4	2	IVIg/CS: good response for CIDP; CS/CIC: partial remission of nephrotic syndrome	Nazarali et al^16^
6-11	Details per patient not provided					IgG4	NA	IVIg: resistant; CS: resistant; PE: resistant; RTX: positive response in 83%	Delmont et al^17^
12	57	M	7.8 g/d	3	NA	IgG1, IgG3	3	Good response to RTX, IVIg, and CS	Xu et al^18^
13	75	M	7.2 g/d	2.8	0.98	IgG4	1	CS/IVIg: partial remission of nephrotic syndrome	Le Quintrec et al^8^
14	71	M	5.7 g/d	2.8	1.73	IgG1, IgG3, IgG4	1	RTX; death	Le Quintrec et al^8^
15	58	M	4.5 g/d	2.7	0.60	IgG4	2	IVIg: partial remission of nephrotic syndrome	Le Quintrec et al^8^
16	80	M	1.8 g/d	2.6	1.13	IgG3, IgG4	1	CS/IVIg/PE; death	Le Quintrec et al^8^
17	66	M	2.2 g/d	2.5	0.93	IgG4	1	CS/IVIg/RTX: complete remission of nephrotic syndrome	Le Quintrec et al^8^
18	61	M	7 g/g Cr	2.1	NA	IgG4	1	IVIg: no response; RTX: complete remission of nephrotic syndrome and neuronal improvement	Plaisier et al^19^
19	37	M	2.9 g/d	NA	NA	IgG4	NA	IVIg: no response; CS/CP: good response on neuropathy	Tan et al^20^
20	65	M	3.5 g/g Cr	NA	Normal	IgG4	NA	CS/IVIG/PE: partial/transient response; RTX: partial remission of nephrotic syndrome	Remiche et al^21^
21	73	F	3.6 g/d	2.9	0.8	IgG4	1	CS: no response; RTX: improvement of neuropathy and nephrotic syndrome	Santoro et al^9^
22	56	M	10 g/g Cr	1.1	NA	IgG4	NA	CS/CP: complete remission of neuropathy; stabilization of nephrotic syndrome	Fehmi et al^22^
23	49	M	6 g/g Cr	2.4	NA	IgG1, IgG4	NA	CS/IVIg: good response on neuropathy, complete remission of nephrotic syndrome	Fehmi et al^22^
24	39	M	3.5 g/d	2.6	NA	IgG4	NA	CS/IVIg/TAC: good response on neuropathy, complete remission of nephrotic syndrome	Fehmi et al^22^
25	50	M	41 g/g Cr	1.2	NA	IgG4	NA	CS/IVIg/PE/CYC/RTX: unresponsive to all; ASCT: good response of neuropathy and nephropathy	Fehmi et al^22^
26	79	M	3 g/d	NA	1.13	N/A	NA	CS/IVIg/PE; death	Fehmi et al^22^
27	74	M	3.4 g/d	3.6	0.98	NA	NA	CS/IVIg/PE/RTX; death	Fehmi et al^22^
28	58	M	10 g/d	NA	1.09	NA	NA	IVIg: mild response; CS: no response; CP/PE; complete remission of neuropathy and nephropathy	Fehmi et al^22^
29	62	M	N/A	1.2	0.76	IgG4	NA	CS/IVIg/PE: unresponsive to all; RTX/CP: good response of neuropathy and nephropathy	Fehmi et al^22^
30	66	M	2.2 g/d	NA	0.93	NA	NA	CS/IVIg/PE/RTX: stabilization of neuropathy and nephropathy	Fehmi et al^22^
31	39	F	NA	NA	NA	NA	NA	CS/IVIg/AZA/MMF: complete remission of neuropathy and stabilization of nephropathy	Fehmi et al^22^
32	52	F	NA	NA	NA	IgG4	NA	CS/IVIg; good response of neuropathy and nephropathy	Fehmi et al^22^
33	60	F	NA	2.7	NA	IgG1, IgG4	NA	CS/IVIg: transient response; RTX; death	Fehmi et al^22^
34	60	M	11.3 g/d	NA	0.95	NA	1	CS/CP: improved	Tang et al^23^
35	46	F	4 g/d	NA	0.61	NA	1	CS: improved	Tang et al^23^
36	14	M	5.5 g/d	NA	0.48	NA	1	CS: improved	Tang et al^23^
37	50	M	0.84 g/g Cr	3.6	0.65	IgG4	3-4	Improved	Shida et al^24^
39	65	M	7.5 g/d	2.1	NA	IgG4	2	CS: partial remission of neuropathy; TAC: complete recovery from right proximal upper extremity weakness; CS/CP: complete remission of nephrotic syndrome	Liu et al^25^
40	45	M	22 g/d	NA	NA	IgG4	2	CS/IVIg/AZA: good response on neuropathy; CIC: partial remission of nephrotic syndrome	Tarabzuni^26^
41	12	M	3.2 g/g Cr	NA	NA	IgG1	2	CS/IVIg: minimal improvement of neuropathy and nephropathy; RTX: further improvement of neuropathy and nephropathy	Bayraktar Eltutan et al^27^
42	70	M	11 g/g Cr	1.5	0.97	NA	2	TAC: insufficient data; IVIg: no response on CIDP; CS/CP/PE/RTX/IVIg; good response of neuropathy, partial remission of nephrotic syndrome	Our study

Abbreviations: ASCT, autologous stem cell transplant; AZA, azathioprine; CIC, ciclosporin; CIDP, chronic inflammatory demyelinating polyneuropathy; CNTN1, contactin-1; CP, cyclophosphamide; CS, corticosteroids; F, female; IgG, immunoglobulin G; IVIg, intravenous immunoglobulin; M, male; MMF, mycophenolate mofetil; MN, membranous nephropathy; NA, not available; PE, plasma exchange; RTX, rituximab; Salb, serum albumin; Scr, serum creatinine; TAC, tacrolimus; Uprot, urinary proteinuria.

Our patient initially presented with PLA2R-negative MN and later developed autoimmune nodopathy. Anti-CNTN1 autoantibodies strongly supported a common antigenic target affecting both podocytes and peripheral nerves. This supports systematic neurological assessment in PLA2R-negative MN, as renal and neurological manifestations may be temporally dissociated. This approach aligns with the Mayo Clinic’s 2-step MN classification, although limited antigen testing availability remains a major limitation.^28^

The pathogenic mechanism of anti-CNTN1 antibodies likely involves immune cross-reactivity, but CNTN1 autoimmunity alone is insufficient, as only a minority of autoimmune nodopathy patients develop MN.^7^^,^^15^^,^^29^ Low-level CNTN1 expression in glomeruli suggests in situ immune complex formation, similar to anti-PLA2R--associated MN.^8^^,^^22^^,^^30^, ^31^, ^32^

Anti-CNTN1--associated MN is predominantly IgG4 mediated.^8^ Although IgG4 is classically considered noninflammatory, complement deposits have been observed in kidney biopsies but not in nerve tissue.^8^^,^^33^ Interestingly, kidney function appears preserved in CNTN1-associated MN compared with other MN subtypes.^34^

Treatment targeted the presumed pathogenic role of anti-CNTN1 antibodies. Standard chronic inflammatory demyelinating polyneuropathy therapies (intravenous Ig and corticosteroids) are often ineffective, with only 20% response rates reported.^22^ In this case, the decision to pursue a combined immunosuppressive approach—including corticosteroids, CP, plasmapheresis,^35^ intravenous Ig, and rituximab—was made to rapidly eliminate circulating antibodies, address their production, and control inflammation. This resulted in rapid neurological improvement and seronegativity. Anti-CNTN1 titers appear to reflect disease activity, similarly to anti-PLA2R antibodies in MN.^19^^,^^22^^,^^36^^,^^37^ The use of intensive combination therapy is supported by prior data in PLA2R-associated MN, showing fast remission while limiting the cumulative dose of CP and steroids.^38^ This is the first case of successful treatment using this immunosuppressive regimen. Another case has been reported in which a similar treatment regimen was used^22^; however, the details and sequence of immunosuppressive therapy remain unknown.

Renal recovery was slower, possibly reflecting delayed glomerular healing. Severe infectious complications occurred after a second treatment course, which may have been unnecessary, given subsequent antibody negativity. Further studies are needed to define optimal treatment strategies and long-term renal outcomes in anti-CNTN1--associated MN.

In conclusion, anti-CNTN1 autoimmunity should be considered in patients with PLA2R-negative MN and neurological symptoms. This case supports a shared antigenic target and highlights the potential benefit—and risks—of aggressive, targeted immunosuppression as the spectrum of MN-associated antigens continues to expand.

## Supplementary Materials

Supplementary File (PDF) Items S1-S3.
