# Supplementary material for: Anti-Contactin-1-Associated Membranous Nephropathy Presenting With Subsequent Autoimmune Nodopathy
Source: Kidney Med. 2026 Jul 18;8(10):101478. doi: 10.1016/j.xkme.2026.101478 (PMC13602309; doi:10.1016/j.xkme.2026.101478)
Supplement: Supplementary file 1 [file mmc1.pdf]

Supplementary Material: Anti-Contactin-1 Associated Membranous Nephropathy Presenting with Subsequent Autoimmune Nodopathy

Item S1. Detailed Methods

Item S2. Neurological examinations

Item S3. Electrophysiological examination of nerves at presentation of autoimmune nodopathy

Item S1. Detailed Methods

Cell-based assay

Sera was tested for antibodies against contactin 1 (CNTN1) using a live, transiently transfected cell-based assay (CBA). For this, human embryonic kidney 293T (HEK) cells were co-transfected with human DNA plasmid constructs for CNTN1 followed by incubation with patient sera. Fluorescently tagged secondary and tertiary antibodies against human IgG (1:1000, anti-goat, R&D #AF904) were used to visualize cell membrane binding by an investigator blind to the sample identity. (S1)

Immunohistochemistry and confocal microscopy of the kidney biopsy

IF staining was performed on FFPE sections retrieved for 30 minutes using target retrieval solution high pH (Dako Target retrieval solution, pH9 ref S2367) in pressure cooker equipment (Bio SB, Santa Barbara, CA). The anti-human CNTN-1 primary antibody (goat polyclonal, R&D systems cat number AF904) was diluted to 1:100 in blocking solution (2% calf fetal serum and 2% normal donkey serum) and incubated overnight at 4 °C with retrieved biopsy sections. Next, the slides were incubated with secondary antibody donkey Alexa 488-conjugated anti-goat IgG (Life Technologies, Thermo Fisher Scientific, Waltham, MA). Finally, slides were mounted in mounted medium (Thermo Fisher Scientific) and covered with LDS2460EP cover glass slides.

Supplementary reference:

S1. Broers MC, Wieske L, Erdag E, Gurlek C, Bunschoten C, van Doorn PA, et al. Clinical relevance of distinguishing autoimmune nodopathies from CIDP: longitudinal assessment in a large cohort. J Neurol Neurosurg Psychiatry. 2023;95(1):52-60.

Item S2. Neurological examinations

Upon presentation of autoimmune nodopathy:

General examination: patient in good general condition, alert and appropriate, mildly pale.

Motor examination: upper extremities — biceps and triceps 5/5 bilaterally; finger flexors 5-/5-; finger extensors 1/4 (right/left); wrist extensors 2/4; wrist flexors 4/4.

Lower extremities — quadriceps 4-/4-, hamstrings 5/5, ankle dorsiflexors 3/3, limited by marked edema.

Sensory examination: distal sensory loss in a length-dependent pattern, with paresthesias in the hands and numbness in the arms, more pronounced distally with proximal improvement. In the lower extremities, sensory loss from above the knees distally, increasing toward the feet.

Coordination: finger–nose testing normal.

Reflexes: symmetrically reduced to absent.

At the start of methylprednisolone, prednisolone, cyclophosphamide, plasmapheresis and rituximab:

Patient is wheelchair-bound. Alert and appropriate.
<u>Cranial nerves:</u> Pupils equal and reactive to light; extraocular movements intact. No ptosis. No facial asymmetry. Facial sensation not tested.
<u>Motor function:</u> Upper extremities: Deltoids bilaterally MRC (right/left) 4/4; biceps 4.5/4.5; triceps 5-/5-; hand grip 3/3; wrist extensors 4/4-; wrist flexors 5-/5-; finger extensors 1/4-; finger abductors 1/3; finger flexors 3/4.
Lower extremities: Iliopsoas 4/4; hamstrings 5-/5-; quadriceps 4/5; ankle dorsiflexors 2/2; plantar flexors 5/5.
<u>Sensation:</u> Hypoalgesia in the arms and hands, most pronounced distally in the hands. Hypoalgesia in the feet and lower legs bilaterally. Vibration sense absent in the hands and lower extremities.
<u>Coordination:</u> Finger-to-nose testing impaired due to weakness.
<u>Reflexes:</u> Triceps reflexes -1/-1; all other reflexes absent; plantar responses indifferent bilaterally.

Marked edema of the feet, ankles, and lower legs.

Patient is able to stand up from the wheelchair with great difficulty but is unable to walk.

Ten months after presentation of autoimmune nodopathy:

Patient ambulates into the room. Alert and appropriate.

Cranial nerves: Pupils equal and reactive to light; extraocular movements intact. No facial asymmetry.

Motor function: Upper extremities: Deltoid muscles bilaterally MRC (right/left) 5/5; biceps bilaterally 5/5; triceps 5/-; grip strength 3/4; wrist extensors 3/4; wrist flexors 5-/5-; finger extensors 1/4-; finger abductors 1/3; finger flexors 3/5-. Lower extremities: Iliopsoas 5-/5-; hamstrings 5-/5-; quadriceps 5/5; ankle dorsiflexors 4/4; plantar flexors 5/5.

Sensation: Not tested.

Reflexes: Triceps reflexes normal bilaterally; all other reflexes absent; plantar responses indifferent bilaterally.

Gait characterized by bilateral foot drop (steppage gait).

Item S3. Electrophysiological examination of nerves at presentation of autoimmune nodopathy

Sensory nerves:

Nerve	Site	DML (ms)	MCV (m/s)	Ref (m/s)	Amplitude (μV)	Ref (μV)
R Ulnar	Digit V	NR	NR	≥45.0	NR	—
L Ulnar	Digit V	NR	NR	≥45.0	NR	—
R Sural	Lat. malleolus	NR	NR	≥39.0	NR	≥4.0
L Sural	Lat. malleolus	NR	NR	≥39.0	NR	≥4.0
R Radial	Snuffbox	4.4	22.6	≥45.0	2.5	—
L Radial	Snuffbox	4.1	24.5	≥45.0	3.7	—

Motor nerves:

Nerve	Muscle	DML (ms)	Ref (ms)	MCV (m/s)	Ref (m/s)	CMAP (mV)	Ref (mV)
R Median	APB	10.8	≤4.5	27.3	≥49.0	1.0	≥5
L Median	APB	9.9	≤4.5	17.4	≥49.0	0.3	≥5
R Ulnar	ADM	7.4	≤3.7	33.3	≥50.0	3.5	≥7.9
L Ulnar	ADM	7.4	≤3.7	24.2	≥50.0	0.2	≥7.9
R Ulnar	FDI	8.4	≤4.6	25.5	≥50.0	2.7	≥5.1
L Ulnar	FDI	9.1	≤4.6	29.8	≥50.0	3.1	≥5.1
R Tibial	AH	8.7	≤6.1	36.4	≥34.8	0.2	≥3.5
L Tibial	AH	4.5	≤6.1	NR	≥34.8	0.0	≥3.5
R Peroneal	EDB	NR	≤6.5	NR	—	NR	—
L Peroneal	EDB	NR	≤6.5	NR	—	NR	—
R Peroneal	TA	7.2	—	15.2	≥37.7	0.6	—
L Peroneal	TA	6.9	—	13.8	≥37.7	0.7	—
R Radial	EIP	6.6	≤2.5	14.5	≥60.0	0.3	—
L Radial	EIP	4.0	≤2.5	21.4	≥60.0	1.2	—

F-waves:

Nerve	F-wave (ms)	Ref (ms)	F–M interval (ms)
R Median	81.5	≤28.2	71.4

L Median	83.0	≤28.2	73.6
R Ulnar	64.1	≤28.4	55.7
L Ulnar	68.4	≤28.4	60.4
Radial (bilat.)	NR	—	—

ADM: abductor digiti minimi; AH: abductor hallucis; APB: abductor pollicis brevis; CMAP: compound muscle action potential; DML: distal motor latency; EDB: extensor digitorum brevis; EIP: extensor indicis proprius; FDI: first dorsal interosseous; MCV, motor nerve conduction velocity; ND: not detected; NR: no response; TA: tibialis anterior.

Conclusion:

According to the EFNS criteria (2021), the findings fulfill the electrodiagnostic criteria for CIDP/demyelinating polyneuropathy, based on prolonged distal motor latencies (DMLs) in the ulnar nerve, reduced motor conduction velocities in the median and ulnar nerves bilaterally, and prolonged F-wave latencies in all four nerves exceeding 20% above the upper limit of normal (ULN).

In addition, there is evidence of extensive and severe sensorimotor axonal loss.
